# Measurement data from bobbins of Partially Oriented Yarns: Univariate and multivariate aspects

**DOI:** 10.1016/j.dib.2019.104637

**Published:** 2019-10-07

**Authors:** Fabrício A. Almeida, Daniel S. Cortez, Guilherme F. Gomes, Juliana H.D. Gaudêncio, Rachel C. Sabioni, José H.F. Gomes, Anderson P. Paiva

**Affiliations:** a Institute of Industrial Engineering and Management, Federal University of Itajubá, Brazil; b Institute of Mechanical Engineering, Federal University of Itajubá, Brazil; c Department of Mechanical Systems Engineering, Sorbonne University, University of Technology of Compiègne, France

**Keywords:** Partially oriented yarns, Measurement data, Automatic package analyser, Univariate and multivariate aspects, Gage repeatability and reproducibility

## Abstract

In this paper, we present data from measurements made in the textured fibers bobbins in two different conditions, presenting critical quality characteristics such as diameter, mass and density. In order to obtain a significant amount of information, in each of the two conditions, 270 measurements were obtained for each of the quality characteristics. Three different equipments (Automatic Package Analyzer - APA) were used in ten different parts, replicated three times for each of them. Considering the two measurement data collection, an amount of 540 bobbins measurements were obtained. Almeida et al., (2019) applied these measurement data in his study. Taking into account the multicorrelated nature of the information, we also have the representation of the principal components' scores for these measurements, besides the eigenvalues and eigenvectors of the data.

**Table:** Specifications Table

Subject area	*Engineering*
More specific subject area	*Industrial Engineering, measurement system analysis, texturing process.*
Type of data	*Table*
How data was acquired	*Automatic Package Analyzer, software.*
Data format	*Measured and Analyzed.*
Experimental factors	*The parts to be analyzed were chosen in a random manner, but presented different characteristics, representing the actual amplitude of the process. Due to the dynamics of the company, each data set differs.*
Experimental features	*The measurements of the parts was performed randomly, in order to avoid bias. Measurements are performed automatically by measurements equipment (Automatic Package Analyzer - APA). The scores of the principal components were generated from the original measurements data.*
Data source location	*Data collected from a textile industry located in southern Minas Gerais, Brazil.*
Data accessibility	*All data are presented in this article*

**Table:** 

**Value of the Data** • These data can be used for several applications in a measurement system analysis (MSA), allowing the application of different techniques or analysis strategies; • There are few works aimed at evaluating measurements in this area, so these data contribute to possible applications and analyzes of methodologies such as the six sigma application in this process. • It is possible to use and analyze both univariate and multivariate strategies from these data, as in gage study. Thus being able to consolidate possible and new methods

## 1 Data

These data present the measurements of a real textured fibers bobbins process of type Partially Oriented Yarns (POY), randomly and automatically collected from specific measurements devices called Automatic Package Analyzer (APA). Data were used in the study by Almeida et al. [1]. The data present a great amount of information, describing three of the main critical characteristics of the quality, such as: diameter (cm), mass (g) and density (g/cm^3^). In manufacturing processes that have many quality characteristics, one must also verify the data's multi-correlation [2], so the principal components' scores of these measurements are also presented, along with their eigenvalues and eigenvectors. The information based on principal components was used due to the great applicability of this strategy, as in works of [3,4]. There are two distinct conditions in this data set, where each of them presents 270 information for the original data and 270 information of the principal components' scores.

Table 1, Table 4 present the original measurement data collected under different analysis conditions (without calibration and after calibration, respectively). The scores of the main components of each data collection are described in Table 2, Table 5, respectively. Finally, in Table 3, Table 6 it is possible to verify the eigenvalues and eigenvectors of the collected data, respectively.

**Table 1:** First measurement data collection.

*n*	*a*	APA1			APA2			APA3		
		Mass	Diameter	Density	Mass	Diameter	Density	Mass	Diameter	Density
		[g]	[cm]	[g/cm³]	[g]	[cm]	[g/cm³]	[g]	[cm]	[g/cm³]
1	1	3336	22.861	0.3994	3334	22.752	0.3890	3340	22.638	0.4031
1	2	3299	21.848	0.4371	3298	21.776	0.4354	3302	21.666	0.4452
1	3	3348	21.17	0.4768	3344	21.142	0.4701	3349	21.126	0.4754
1	4	3327	21.177	0.4717	3327	21.082	0.4703	3332	20.975	0.4794
1	5	3328	22.012	0.4345	3325	21.915	0.4338	3330	21.774	0.4433
1	6	3315	21.993	0.4322	3314	21.873	0.4330	3318	21.785	0.4393
1	7	3342	21.233	0.4723	3337	21.116	0.4699	3342	20.968	0.4845
1	8	3339	22.313	0.4220	3336	22.187	0.4257	3341	22.105	0.4273
1	9	3333	21.46	0.4565	3336	21.416	0.4526	3341	21.432	0.4576
1	10	3330	22.321	0.4227	3330	22.191	0.4228	3336	22.091	0.4313
2	1	3337	22.805	0.4025	3336	22.729	0.3969	3341	22.638	0.4056
2	2	3301	21.881	0.4359	3298	21.792	0.4339	3302	21.724	0.4407
2	3	3344	21.177	0.4659	3343	21.149	0.4711	3349	21.115	0.4744
2	4	3328	21.147	0.4697	3326	21.058	0.4711	3331	21.04	0.4740
2	5	3328	21.956	0.4383	3326	21.866	0.4301	3331	21.76	0.4446
2	6	3311	21.963	0.4320	3314	21.838	0.4282	3318	21.788	0.4388
2	7	3340	21.177	0.4706	3336	21.092	0.4766	3342	21.022	0.4787
2	8	3337	22.306	0.4244	3336	22.214	0.4232	3340	22.087	0.4305
2	9	3338	21.505	0.4539	3335	21.42	0.4565	3341	21.367	0.4593
2	10	3334	22.332	0.4243	3330	22.173	0.4209	3336	22.119	0.4291
3	1	3335	22.857	0.3977	3335	22.758	0.3975	3340	22.638	0.4057
3	2	3300	21.855	0.4369	3298	21.812	0.4312	3302	21.68	0.4419
3	3	3345	21.241	0.4716	3342	21.177	0.4655	3349	21.022	0.4828
3	4	3329	21.173	0.4712	3327	21.032	0.4742	3332	20.982	0.4802
3	5	3329	22.012	0.4349	3325	21.857	0.4341	3331	21.742	0.4422
3	6	3312	21.941	0.4331	3313	21.85	0.4333	3318	21.792	0.4390
3	7	3339	21.185	0.4713	3337	21.096	0.4688	3343	21.072	0.4756
3	8	3337	22.261	0.4277	3336	22.184	0.4230	3341	22.09	0.4310
3	9	3335	21.516	0.4501	3336	21.402	0.4609	3341	21.367	0.4584
3	10	3332	22.328	0.4222	3331	22.186	0.4236	3336	22.134	0.4290

**Table 2:** Scores for the first measurement data collection.

*n*	a	APA1			APA2			APA3		
		PC_1_	PC_2_	PC_3_	PC_1_	PC_2_	PC_3_	PC_1_	PC_2_	PC_3_
1	1	−2.5957	1.0960	−0.1257	−2.7898	0.9561	0.3122	−2.1314	1.2806	0.0768
1	2	−0.8878	−2.2219	−0.1062	−0.8630	−2.3162	0.0332	−0.3679	−2.1158	−0.0922
1	3	2.0020	0.9055	−0.1338	1.7749	0.6331	0.0779	2.0375	0.9678	−0.0311
1	4	1.4690	−0.6096	−0.1039	1.5481	−0.6409	0.0639	2.0379	−0.3625	−0.0402
1	5	−0.6466	−0.0118	−0.0942	−0.5976	−0.2681	0.0384	−0.0561	−0.0050	−0.0297
1	6	−0.9248	−0.9629	−0.0676	−0.7657	−1.0894	0.0597	−0.4000	−0.8626	0.0095
1	7	1.6838	0.5127	−0.1146	1.6765	0.1092	0.0807	2.3749	0.3439	−0.1304
1	8	−1.1924	0.9812	−0.0603	−0.9790	0.6910	−0.0208	−0.7394	1.0173	0.0669
1	9	0.7790	0.0220	0.0064	0.7765	0.2440	0.1957	0.9915	0.5928	0.0517
1	10	−1.3440	0.3195	−0.1396	−1.1754	0.2662	0.0279	−0.6967	0.6244	−0.0594
2	1	−2.4172	1.1313	−0.1386	−2.4990	1.0547	0.1210	−2.0412	1.3416	0.0079
2	2	−0.9286	−2.0555	−0.1032	−0.9273	−2.3021	0.0573	−0.5706	−2.0700	−0.0359
2	3	1.6089	0.6683	0.1568	1.7764	0.5576	0.0343	2.0237	0.9681	0.0119
2	4	1.4666	−0.5381	0.0010	1.5853	−0.7284	0.0647	1.7806	−0.3826	0.0301
2	5	−0.4679	−0.0531	−0.1310	−0.6250	−0.1959	0.2189	0.0196	0.0559	−0.0470
2	6	−0.9645	−1.2681	−0.0434	−0.8589	−1.0798	0.2472	−0.4182	−0.8589	0.0204
2	7	1.6705	0.3513	−0.0017	1.8790	−0.0069	−0.0884	2.1379	0.3949	−0.0283
2	8	−1.1501	0.8194	−0.1331	−1.0842	0.7143	0.0166	−0.6427	0.9207	−0.0091
2	9	0.7379	0.4204	0.0496	0.8667	0.1526	0.0686	1.1228	0.5580	0.0871
2	10	−1.2395	0.6099	−0.1807	−1.2063	0.2681	0.1069	−0.7945	0.6465	−0.0322
3	1	−2.6558	1.0291	−0.0772	−2.5364	0.9900	0.0596	−2.0566	1.2677	−0.0001
3	2	−0.8838	−2.1446	−0.1049	−1.0298	−2.2806	0.1103	−0.4802	−2.0938	−0.0135
3	3	1.7095	0.7395	−0.0906	1.5603	0.5236	0.1592	2.3809	0.8890	−0.1116
3	4	1.4938	−0.4614	−0.0713	1.7255	−0.6809	0.0126	2.0512	−0.3635	−0.0722
3	5	−0.6174	0.0598	−0.1003	−0.5147	−0.2932	0.1053	−0.0266	0.0606	0.0477
3	6	−0.8865	−1.2090	−0.0421	−0.7455	−1.1738	0.0754	−0.4181	−0.8582	0.0098
3	7	1.6644	0.2773	−0.0402	1.6696	0.1066	0.1402	2.0037	0.5040	0.0024
3	8	−0.9975	0.7846	−0.1720	−1.0530	0.7033	0.0632	−0.6117	0.9925	−0.0251
3	9	0.5593	0.2236	0.1330	1.0347	0.1968	−0.0331	1.0983	0.5622	0.1123
3	10	−1.3314	0.4718	−0.1233	−1.1294	0.3339	0.0174	−0.8157	0.6529	−0.0498

**Table 3:** Eigenvalues and eigenvector for the first measurement data collection.

	PC_1_	PC_2_	PC_3_
Eigenvalue	2.0491	0.9416	0.0094
Proportion	0.683	0.314	0.003
Cumulative	0.683	0.997	1
CTQ's	Eigenvectors		
Mass	0.238	0.969	0.069
Diameter	−0.681	0.217	−0.699
Density	0.692	−0.12	−0.712

**Table 4:** Second measurement data collection.

*n*	*a*	APA1			APA2			APA3		
		Mass	Diameter	Density	Mass	Diameter	Density	Mass	Diameter	Density
		[g]	[cm]	[g/cm³]	[g]	[cm]	[g/cm³]	[g]	[cm]	[g/cm³]
1	1	3475	21.923	0.4502	3473	21.744	0.4507	3478	21.726	0.4526
1	2	3453	21.091	0.4861	3452	20.967	0.4871	3457	20.957	0.4873
1	3	3475	22.062	0.4430	3477	21.903	0.4443	3480	21.892	0.4432
1	4	3477	21.613	0.4642	3478	21.499	0.4647	3480	21.5	0.4652
1	5	3470	21.534	0.4663	3469	21.402	0.4674	3473	21.456	0.4662
1	6	3492	22.868	0.4098	3489	22.784	0.4099	3496	22.728	0.4109
1	7	3474	21.97	0.4470	3473	21.821	0.4476	3479	21.815	0.4484
1	8	3457	22.195	0.4338	3458	22.057	0.4346	3460	22.027	0.4356
1	9	3473	22.642	0.4166	3473	22.494	0.4175	3477	22.444	0.4181
1	10	3475	22.119	0.4423	3474	21.999	0.4414	3478	21.991	0.4424
2	1	3475	21.882	0.4516	3475	21.748	0.4515	3478	21.725	0.4518
2	2	3454	21.099	0.4853	3451	20.982	0.4855	3457	20.95	0.4878
2	3	3477	22.062	0.4413	3477	21.898	0.4440	3481	21.896	0.4443
2	4	3479	21.587	0.4647	3477	21.408	0.4648	3481	21.449	0.4652
2	5	3467	21.542	0.4665	3469	21.45	0.4653	3473	21.427	0.4671
2	6	3492	22.907	0.4094	3493	22.78	0.4093	3496	22.75	0.4109
2	7	3474	21.907	0.4468	3475	21.836	0.4468	3478	21.789	0.4484
2	8	3457	22.17	0.4348	3456	22.054	0.4350	3460	22.06	0.4352
2	9	3473	22.606	0.4166	3474	22.485	0.4173	3476	22.493	0.4183
2	10	3475	22.114	0.4417	3476	22.033	0.4413	3480	21.981	0.4432
3	1	3475	21.878	0.4521	3475	21.766	0.4503	3478	21.717	0.4522
3	2	3452	21.096	0.4871	3452	21.01	0.4855	3457	20.939	0.4872
3	3	3477	22.041	0.4431	3478	21.926	0.4430	3482	21.849	0.4455
3	4	3477	21.581	0.4648	3475	21.473	0.4652	3481	21.473	0.4653
3	5	3469	21.525	0.4665	3470	21.425	0.4661	3473	21.42	0.4673
3	6	3492	22.891	0.4100	3489	22.784	0.4089	3496	22.743	0.4113
3	7	3473	21.967	0.4456	3475	21.834	0.4465	3477	21.755	0.4495
3	8	3456	22.18	0.4336	3457	22.057	0.4341	3461	22.056	0.4350
3	9	3473	22.661	0.4164	3471	22.516	0.4167	3477	22.51	0.4172
3	10	3478	22.128	0.4406	3474	21.984	0.4416	3480	21.959	0.4425

**Table 5:** Scores for the second measurement data collection.

*n*	a	APA1			APA2			APA3		
		PC_1_	PC_2_	PC_3_	PC_1_	PC_2_	PC_3_	PC_1_	PC_2_	PC_3_
1	1	−0.0069	−0.1885	0.1416	−0.3349	−0.1487	−0.0925	−0.1785	−0.6023	−0.0732
1	2	−3.0601	0.4647	0.1627	−3.2877	0.4496	0.0201	−3.0738	0.0301	−0.0033
1	3	0.3664	0.0239	0.1112	0.2262	−0.2647	−0.0812	0.3825	−0.4988	−0.1413
1	4	−0.6892	−0.7911	0.1377	−0.7977	−0.9551	−0.0127	−0.7178	−1.1268	−0.0015
1	5	−1.1702	−0.3044	0.1132	−1.4105	−0.3265	−0.0376	−1.1247	−0.5988	−0.0109
1	6	3.0800	−0.2826	0.1544	2.8343	−0.0928	0.0467	3.0616	−0.7194	−0.0233
1	7	0.0944	−0.0217	0.1100	−0.1529	−0.0462	−0.0810	0.0954	−0.5552	−0.0827
1	8	−0.0462	1.7411	0.0643	−0.1928	1.5565	−0.1108	−0.1652	1.3562	−0.1281
1	9	1.7295	1.0121	0.1065	1.5214	0.9012	−0.0766	1.6282	0.5311	−0.1415
1	10	0.4564	0.0726	0.1705	0.2870	0.0924	−0.0269	0.4345	−0.2574	−0.0189
2	1	−0.0968	−0.2389	0.1275	−0.2596	−0.3236	−0.0675	−0.1573	−0.5893	−0.1001
2	2	−2.9814	0.4016	0.1455	−3.2707	0.5685	−0.0063	−3.0965	0.0171	0.0026
2	3	0.5067	−0.1110	0.0508	0.2284	−0.2628	−0.0979	0.4030	−0.5969	−0.1036
2	4	−0.6426	−0.9802	0.1102	−0.9594	−0.9336	−0.1366	−0.7344	−1.2416	−0.0776
2	5	−1.3050	−0.0569	0.1404	−1.2924	−0.2597	−0.0358	−1.1857	−0.6329	−0.0237
2	6	3.1394	−0.2506	0.1975	3.0316	−0.4128	0.0095	3.0888	−0.7052	0.0082
2	7	0.0221	−0.0590	0.0135	−0.0193	−0.1867	−0.0912	0.0169	−0.4901	−0.1168
2	8	−0.1051	1.7079	0.0604	−0.3004	1.7116	−0.0962	−0.1132	1.3843	−0.0936
2	9	1.6850	0.9888	0.0550	1.5622	0.8169	−0.0990	1.6368	0.6412	−0.0619
2	10	0.4670	0.0796	0.1442	0.4245	−0.0478	0.0124	0.4925	−0.4414	−0.0139
3	1	−0.1157	−0.2500	0.1377	−0.2038	−0.2916	−0.0799	−0.1784	−0.6013	−0.0988
3	2	−3.1282	0.5328	0.2048	−3.1897	0.5046	0.0307	−3.0933	0.0202	−0.0323
3	3	0.4304	−0.1552	0.0781	0.3374	−0.3096	−0.0928	0.3577	−0.7296	−0.1358
3	4	−0.7456	−0.8220	0.1110	−0.9830	−0.7346	−0.0246	−0.7076	−1.2279	−0.0401
3	5	−1.2333	−0.2317	0.1098	−1.2993	−0.3714	−0.0492	−1.2000	−0.6408	−0.0274
3	6	3.1028	−0.2712	0.1937	2.8623	−0.0757	0.0148	3.0690	−0.7165	0.0109
3	7	0.0835	0.0821	0.0642	−0.0134	−0.1829	−0.1036	−0.1022	−0.4488	−0.1274
3	8	−0.1055	1.8167	0.0396	−0.2252	1.6469	−0.1236	−0.0662	1.3033	−0.1088
3	9	1.7585	1.0277	0.1274	1.4782	1.0929	−0.0644	1.7350	0.5890	−0.0757
3	10	0.6542	−0.1384	0.1199	0.2628	0.0794	−0.0419	0.4849	−0.4436	−0.0677

**Table 6:** Eigenvalues and eigenvector for the second measurement data collection.

	PC_1_	PC_2_	PC_3_
Eigenvalue	24.555	0.5352	0.0093
Proportion	0.819	0.178	0.003
Cumulative	0.819	0.997	1
CTQ's	Eigenvectors		
Mass	0.492	−0.87	−0.033
Diameter	0.619	0.323	0.716
Density	−0.612	−0.373	0.697

## 2 Experimental design, materials, and methods

The data collection was carried out in a textile company that is the market leader in Brazil, where the textured fibers bobbins process represents 15% of the total production of the factory. To ensure a satisfactory amount of data, the information can be divided into two measurement data collection. In accordance with the requirements suggested by the AIAG [5], in both studies, the measurements of ten distinct parts was collected for three different operators and three replicates were carried out, characterizing in a quantity satisfactory information for applications of measurement techniques. This data collection methodology is widely used in measurement system analysis studies, such as [6,7]. It is important to note that the collection was performed in a random manner. From these data, the data of the scores of the principal components' scores were extracted.

In the first data, the measurements of the original data are shown in Table 1 and the principal components' scores of these data are shown in Table 2. Table 3 presents the eigenvalues and eigenvector data of the components.

In view of the second data collection, the Table 4 presents the original data of the bobbin's measurements. The dimensionless data for the components' scores are shown in Table 5. Table 6 describes the eigenvalue and eigenvector data for the scores, representing the second measurement data collection.
